# A Novel *in situ* Approach to Studying Pancreatic Ducts in Mice

**DOI:** 10.3389/fphys.2019.00938

**Published:** 2019-07-24

**Authors:** Eleonóra Gál, Jurij Dolenšek, Andraž Stožer, Viljem Pohorec, Attila Ébert, Viktória Venglovecz

**Affiliations:** ^1^Department of Pharmacology and Pharmacotherapy, University of Szeged, Szeged, Hungary; ^2^Faculty of Medicine, University of Maribor, Maribor, Slovenia; ^3^Faculty of Natural Sciences and Mathematics, University of Maribor, Maribor, Slovenia

**Keywords:** pancreas, slice, duct, calcium, CFTR, Giemsa, chenodeoxycholic acid

## Abstract

**Introduction:** The tissue slice technique offers several benefits compared to isolated cells and cell clusters that help us understand the (patho)physiology of several organs *in situ*. The most prominent features are preserved architecture and function, with intact homotypic and heterotypic interactions between cells in slices. In the pancreas, this technique has been utilized successfully to study acinar and endocrine islet cells. However, it has never been used to investigate ductal function. Since pancreatic ductal epithelial cells (PDECs) play an essential role in the physiology of the pancreas, our aim was to use this technique to study PDEC structure and function *in situ*.

**Materials and methods:** Eight- to sixteen weeks old C57BL/6 mice were used for preparation of pancreas tissue slices. Low melting point agarose was injected into the common bile duct and the whole organ was extracted. For morphological studies, pieces of tissue were embedded in agarose and cryosectioned to obtain 15 μm thick slices. In order to visualize pancreatic ducts, (i) the Giemsa dye was added to the agarose and visualized using light microscopy or (ii) immunostaining for the cystic fibrosis transmembrane conductance regulator (CFTR) was performed. For functional characterization, agarose-embedded tissue was immediately cut to 140 μm thick tissue slices that were loaded with the cell permeant form of the Oregon Green 488 BAPTA-1 dye and used for confocal calcium imaging.

**Results:** Giemsa staining has shown that the injected agarose reaches the head and body of the pancreas to a greater extent than the tail, without disrupting the tissue architecture. Strong CFTR expression was detected at the apical membranes of PDECs and acinar cells, whereas islet cells were completely negative for CFTR. Stimulation with chenodeoxycholic acid (CDCA, 1 mM) resulted in a robust transient increase in intracellular calcium concentration that was readily visible in >40 ductal cells per slice.

**Conclusion:** Our results confirm that the acutely-isolated pancreas tissue slice technique is suitable for structural and functional investigation of PDECs and their relationship with other cell types, such as acini and endocrine cells *in situ*. In combination with different genetic, pharmacological or dietary approaches it could become a method of choice in the foreseeable future.

## Introduction

The tissue slice technique is a suitable *in situ* experimental system for investigating structure and function of different tissues, such as the brain, liver, adrenal gland, and retina (Skrede and Westgaard, 1971; Moser and Neher, 1997; Enoki et al., 2006; Graaf et al., 2007). Speier et al. applied and optimized this technique to study pancreatic beta cell function (Speier and Rupnik, 2003). Since then, it was successfully used to study the functional organization and calcium dynamics of beta cells within islets (Dolenšek et al., 2013; Stožer et al., 2013a,b). The technique has also been applied to characterize acinar cell morphology and secretory function (Marciniak et al., 2013, 2014; Liang et al., 2017). Although there are several *in vitro* approaches for the isolation of both islets and acini from the pancreas, importantly, the greatest advantages of tissue slice preparation technique is that it does not require enzymatic digestion and the architecture and viability of the cells are retained in an intact, nearly physiological environment. It is also important to emphasize that this technique is suitable for both morphological and functional imaging, as well as for electrophysiological studies and investigating interactions between neighboring cells or between the exocrine and endocrine part of the pancreas (Marciniak et al., 2013; Klemen et al., 2014).

Basically, there are two major cell types in the exocrine pancreas, the acinar cells and the pancreatic ductal epithelial cells (PDECs). Although PDECs comprise only a very small fraction of the entire organ, they play an essential role in maintaining the integrity of the pancreas. PDECs secrete an ${\text{HCO}}_{3}^{-}$-rich, alkaline solution that neutralizes the acidic pH of gastric juice, curtails premature trypsinogen activation, and delivers digestive enzymes from the pancreas to the small intestine (Argent Be, 1994; Argent, 2006; Dolensek et al., 2017). Insufficient or decreased ${\text{HCO}}_{3}^{-}$-secretion can lead to cystic fibrosis or trigger acute or chronic pancreatitis (Scheele et al., 1996; Venglovecz et al., 2008, 2011; Hegyi and Rakonczay, 2010). Therefore, intensive research has been conducted to characterize the ductal function both under physiological and pathophysiological conditions (Hegyi et al., 2011; Judák et al., 2014; Katona et al., 2016; Venglovecz et al., 2018). In the 80's, Barry Argent and his colleagues worked out a novel technique that allows the isolation of intact intra-interlobular pancreatic ducts from the pancreas of rodents (Argent et al., 1986). This methodological development was a very important milestone in the physiology of the pancreas, since it pointed out that ductal cells not only provide a framework for acini, but also secrete ${\text{HCO}}_{3}^{-}$. However, this technique has many limitations. Ducts are isolated from the pancreas after an enzymatic digestion that may result in functional changes. The isolation procedure is long and the ducts should be incubated overnight in order to facilitate their regeneration, similarly to isolation and cultivation of islets of Langerhans, which can importantly affect their function (Gilon et al., 1994). In addition, the biggest disadvantage of this technique is that the ductal cells are isolated from their normal environment and, therefore not influenced by other cell types, which can fundamentally support and influence their function. We strongly believe that the pancreas slice preparation is much closer to the physiological conditions than the duct isolation technique and therefore provides a better experimental model to study the function of PDECs both under physiological and pathophysiological conditions.

Our aim in this study was to use the acutely-isolated pancreas tissue slice technique for the morphological and functional investigation of PDECs. We have shown that the ductal cells preserve their viability after the preparation and that the technique is suitable for functional multicellular calcium imaging.

## Materials and Methods

### Ethical Approval

Animal experiments were conducted in compliance with the *Guide for the Care and Use of Laboratory Animals* (United States, Department of Health and Human Services, NIH publication No 85-23, revised 1985) and the experimental protocol was approved by the local Ethical Board of the University of Szeged, the National Scientific Ethical Committee on Animal Experimentation (Budapest, Hungary), and the Veterinary administration of the Republic of Slovenia (permit number: U34401-12/2015/3).

### Chemicals and Solutions

Cystic fibrosis transmembrane conductance regulator (CFTR) human, polyclonal antibody was ordered from Alomone Labs (Jerusalem, Israel). Alexa Fluor Goat Anti-Rabbit IgG secondary antibody was obtained from Abcam (Cambridge, UK). Cell permeant acetoxymethyl ester of Oregon Green 488 BAPTA-1 (OGB-1) was from Invitrogen (Eugene, OR, USA). All other laboratory chemicals were ordered from Sigma-Aldrich Kft. (Budapest, Hungary). Extracellular solution (ECS) contained (in mM): 125 NaCl, 2.5 KCl, 26 NaHCO_3_, 1.25 NaH_2_PO_4_, 6 glucose, 6 lactic acid, 3 myo-inositol, 0.5 ascorbic acid, 2 Na-pyruvate, 1 MgCl_2_, and 2 CaCl_2_. ECS was gassed with 95% O_2_/5% CO_2_ to set pH to 7.4. For calcium dye loading, we used a HEPES-buffered solution containing (in mM): 150 NaCl, 10 HEPES, 6 glucose, 5 KCl, 2 CaCl_2_, 1 MgCl_2_; titrated to pH = 7.4 using 1 M NaOH. For stimulation of PDECs during confocal imaging, we used 1 mM chenodeoxycholic acid (CDCA) dissolved in ECS.

### Preparation of Pancreas Tissue Slices

**Figure 1:**
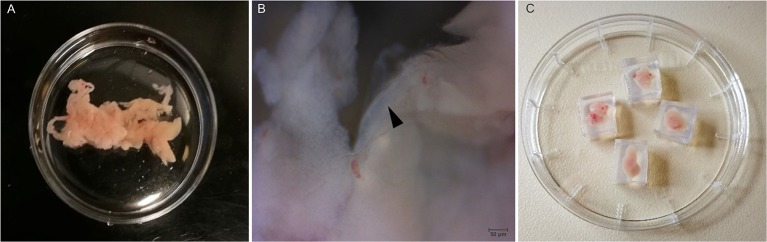
Preparation of pancreas slices. **(A)** Mouse pancreas after injection with agarose. **(B)** Higher magnification (40X) of the isolated pancreas with an intralobular duct (black arrow head). **(C)** Pancreas tissue pieces embedded in agarose cubes.

Eight- to Sixteen weeks old C57BL/6 mice of either sex were used. Preparation of acutely-isolated pancreas tissue slices has been described in detail previously (Speier and Rupnik, 2003; Stožer et al., 2013a; Marciniak et al., 2014). Briefly, after sacrificing the animal, the abdomen was accessed via median laparatomy, the papilla of Vater clamped distally and 1.5–2.5 ml of low-melting-point agarose (1.5–1.8%, with or without Giemsa dye, according to protocol) was injected into the common bile duct using 30 G needles. The injected pancreas was then cooled with ice-cold ECS and transferred into a sterile Petri dish containing ice-cold ECS (Figure 1A). Higher magnification image of the gland shows the presence of an interlobular duct (Figure 1B). In the next step, the pancreas was cleaned from fat and connective tissue, and cut into small pieces (0.25–1.0 cm^3^ in size) using surgical scissors. Individual pieces of agarose-injected pancreas were embedded in agarose (1.5–1.8%) (Figure 1C) and further sectioned either for immunohistochemistry (IHC) or for calcium imaging. For IHC, the isolated tissue was further embedded in cryomatrix and cut into 15 μm sections using a CM1800 cryostat (Leica Biosystems, Wetzlar, Germany). For calcium imaging, 140 μm thick sections were cut using a VT1000 vibratome (Leica Biosystems, Wetzlar, Germany) while the tissue was continuously buffered with ice-cold gassed ECS. Thirty to forty slices were prepared per animal and used immediately for staining.

### Immunohistochemistry

The cryosections were fixed in 4% (v/v) paraformaldehyde for 20 min at room temperature (RT) and washed in PBS 2–3 times. Slices were permeabilized with 0.05% TritonX-100 at RT for 30 min and blocked with the mixture of 1% (v/v) bovine serum albumin/Tris-buffered saline (BSA/TBS) and 10% (v/v) goat serum for 30 min. After the blocking step, slices were incubated with the CFTR rabbit polyclonal antibody (1:100 dilutions) at 4°C, overnight. After the incubation, slices were washed 2–3 times with PBS and incubated with Alexa fluor 488-conjugated goat anti-rabbit IgG secondary antibody (1:400 dilutions) for 3 h at RT. Nuclei were stained with DAPI (1:500 dilutions in BSA/TBS) for 15 min, followed by washing three times in PBS. Slices were mounted using Fluoromount and analyzed using a LSM 880 confocal laser scanning microscope (Carl Zeiss Technika Kft., Budaörs, Hungary). Pancreas slices were excited at 405 (Dapi) and 488 (Alexa fluor 488) nm and emissions were collected at 453 and 516 nm, respectively.

### Giemsa Staining

Giemsa was diluted in low-melting-point agarose (1.5%) at a ratio of 1:10, then injected into the common bile duct of the mice as described in the Preparation of Pancreas Tissue Slices section. After the injection, the pancreas was removed cleaned and cut into three pieces (head, body, and tail). Each pieces of the pancreas were then embedded into cryomatrix and cut into 15 μm sections using a CM1800 cryostat (Leica Biosystems, Wetzlar, Germany) and Giemsa staining was analyzed using an Axio Scope.A1 light microscope (Carl Zeiss Technika Kft., Budaörs, Hungary).

### Calcium Imaging

Ten to fifteen slices were incubated in dye-loading solution containing 6 μM of OGB-1, 0.03% Pluronic F-127 (w/v) and 0.12% dimethylsulphoxide (DMSO, v/v, dissolved in HEPES-buffered solution) for 50 min at RT on an orbital shaker (50 turns min^−1^). Imaging was made within 12 h after staining. Following staining, slices were kept protected from light in a dye-free HEPES-buffered solution, which was exchanged every 2 h. Individual slices were transferred into the recording chamber of either a Leica TCS SP5 II inverted confocal system [Leica HCX PL APO CS 20x immersion objective (NA = 0.7)] or an upright Leica TCS SP5 II confocal system [Leica HCX APO L water immersion objective (20x, NA = 1.0)]. Slices were continuously perfused with gassed ECS at 37°C. OGB-1 was excited by an argon 488 nm laser and fluorescence detected by Leica HyD hybrid detector in the range of 500–650 nm. Eight-bit 512 × 512 pixels images at 1 frame per second were acquired. CDCA stimulation was achieved by manually exchanging delivery tubes of the perifusion system.

## Results

### Visualization of Pancreatic Ducts in Freshly Prepared Slices

**Figure 2:**
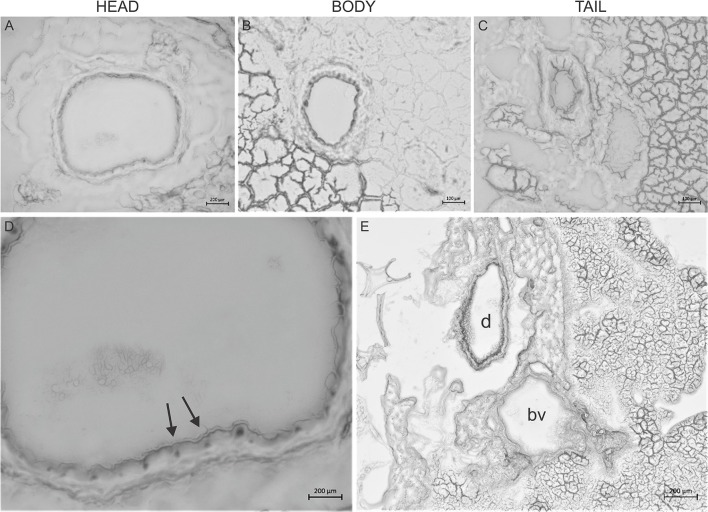
Giemsa staining of the pancreas. Representative cryosections were cut from the head **(A)**, body **(B)**, and tail **(C)** of the pancreas. Giemsa stain causes dark coloring of the nuclei of inter-intralobular ducts in the head and body of the pancreas and slightly in the tail. **(D)** Magnified picture of **(A)**. Arrows indicate dark coloring of the nuclei. **(E)** Representative cryosection from the head of the pancreas shows that Giemsa stained the duct (d) but not the blood vessels (bv).

**Figure 3:**
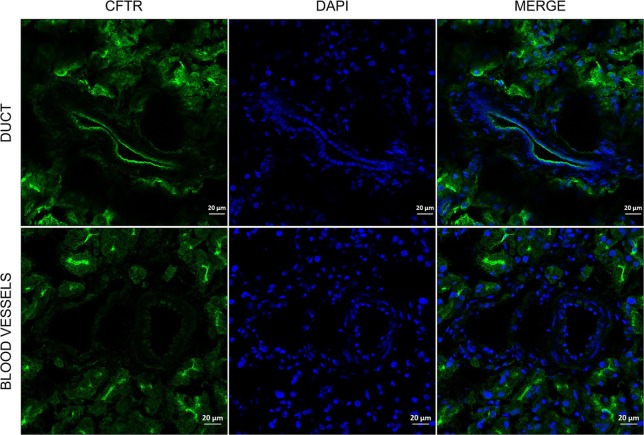
Representative immunofluorescence staining of CFTR in agarose-injected mice pancreas. Pictures were taken at 20X magnification.

In order to investigate how deep, the agarose penetrates into the ductal tree, Giemsa dye was added to the low melting point agarose (1.5%) in 1:10 dilutions and injected into the main pancreatic duct, as described in section Materials and Methods. Freshly prepared pancreas slices of 15 μm thickness from the head, body, and the tail were examined under a stereomicroscope. Figure 2 shows representative tissue slices with intact pancreas morphology and visible structures of exocrine and endocrine cells. Strong nuclei staining was detected in the intra- and inter-lobular ducts of the head (Figures 2A,B) and the body of the pancreas (Figure 2C), whereas only weak staining was found in the tail part (Figure 2D). Blood vessels were completely negative (Figure 2E), indicating that the injection affects only the ductal tree. Using CFTR immunostaining, we were able to identify more specifically the ductal cells in the tissue slices. Under normal conditions, CFTR channel is expressed at the apical membrane of the ductal cells. As shown on Figure 3, strong CFTR staining was detected at the apical membrane of PDECs and acini, whereas islet cells and blood vessels were completely negative for CFTR.

### CDCA Stimulation Induced a Transient Change in Intracellular Calcium Concentration

**Figure 4:**
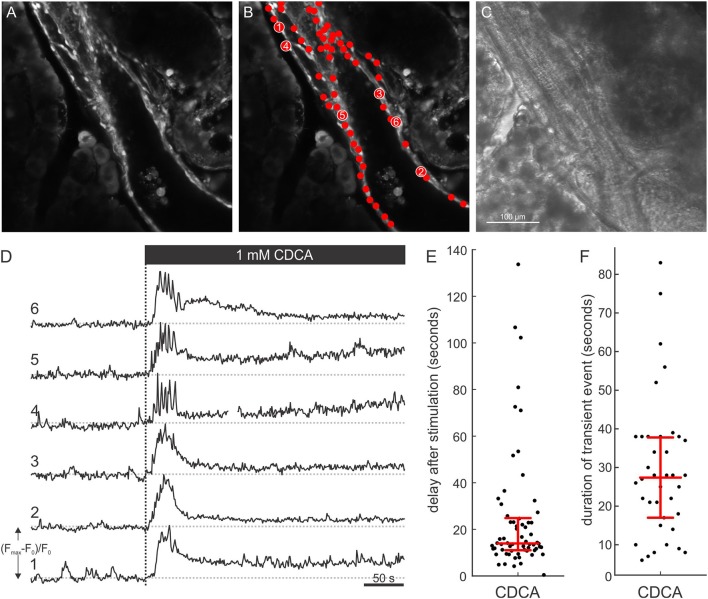
[Ca^2+^]_i_ response in ductal cells after stimulation with 1 mM CDCA. **(A)** Confocal fluorescence image of a ductal structure and surrounding acinar tissue with selected regions of interest (ROI) denoting nuclei of individual PDECs. **(B)** Chenodeoxycholic acid (CDCA) induced [Ca^2+^]_i_ responses of individual cells corresponding to numbered ROIs in **(B)** (1–6). **(C)** Concurrent transmitted light image. **(D)** Dashed vertical line indicates the start of exposure to 1 mM CDCA. **(E,F)** Bee swarm plots with interquartile ranges and medians for delays of responses after stimulation with 1 mM CDCA (*n* = 65 cells from 4 slices) **(E)** and durations of transient [Ca^2+^]_i_ events (*n* = 38 cells from 4 slices) **(F)**.

To functionally characterize ductal-like structures, we resorted to confocal calcium imaging. Under non-stimulatory conditions, PDECs were brightly stained with OGB-1. Moreover, the dye accumulated in the nuclei producing a typical visual pattern of a mono-layered epithelium (Figures 4A–C and Supplemental Video 1). The tissue slice technique also enables simultaneous visualization of exocrine acinar cells and islets of Langerhans (Supplemental Figure 1 and Supplemental Video 1). Moreover, calcium activity could be recorded from acinar cells in parallel with PDEC activity, whereas the islets of Langerhans did not respond to the stimulus used in this study (Supplemental Figure 2). We stimulated the PDECs using a square pulse-like protocol in which the tissue slices were initially perifused with ECS only, followed by ECS containing 1 mM CDCA for 10 min. CDCA stimulation evoked a response that was detected in many cells within a single visual field (Figure 4B). Individual PDECs responded with a transient increase in [Ca^2+^]_i_, followed by a decrease in [Ca^2+^]_i_ to a sustained plateau (Figure 4D). The median response delay to CDCA stimulation was 14.0 s (Q1 = 11.0 s and Q3 = 24.8 s, Figure 4E) and the median duration of the transient change in [Ca^2+^]_i_ was 27.5 s (Q1 = 17.0 s and Q3 = 38.0 s, Figure 4F).

**Figure 5:**
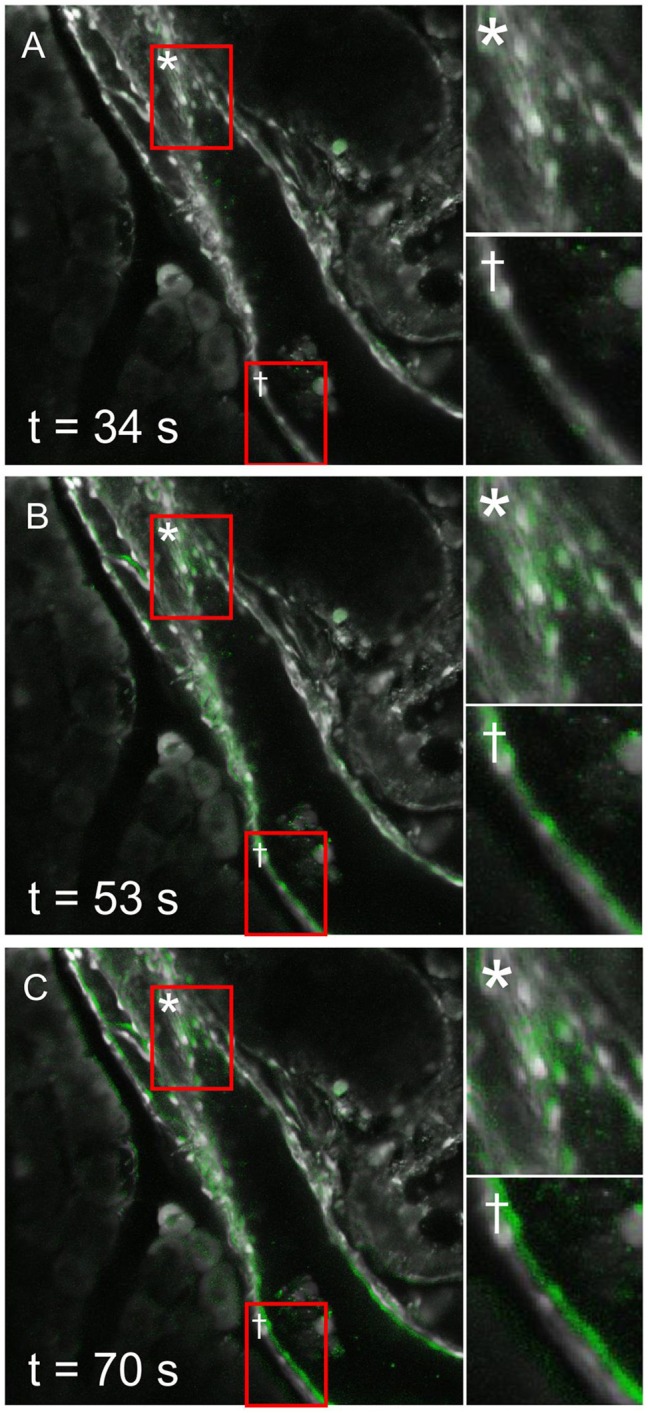
Spatial displacement of ductal cells after stimulation with 1 mM CDCA. An intralobular duct is shown before **(A)** and during **(B,C)** the stimulation with chenodeoxycholic acid (CDCA). Constructs depicting early **(B)** and late **(C)** [Ca^2+^]_i_ response to CDCA display a background of cellular morphology in gray, and an overlay of calcium response in green (see Results section for detailed description). In some PDECs no spatial movement was detected (insets *), whereas in some cells noticeable spatial displacements were observed (insets ^*†*^). Please note that in the PDECs that were displaced during CDCA response, change in [Ca^2+^]_i_ preceded the spatial displacement, therefore not interfering with the calcium response detection.

Coupled with the calcium response, a spatial displacement of PDECs was observed upon stimulation with CDCA (Figure 5). In order to exclude the possibility that the detected [Ca^2+^]_i_ signal in response to CDCA stimulation was an artifact of cell displacement, we meticulously characterized the movement and compared it with the [Ca^2+^]_i_ signal. The calcium response was calculated as *Fresp*(*t*) = *F*(*t*)−*F*_0_, where *Fresp*(*t*) presents the calculated calcium response at time *t*, *F*_0_ the average of the first 100 frames under non-stimulatory conditions and *F*(*t*) the calcium signal at time *t*. A construct was created in which the *F*_0_ frame was displayed in grayscale and *Fresp*(*t*) was overlaid in green (Figure 5 and Supplemental Video 2). Figure 5 shows this construct before CDCA stimulation (Figure 5A), as well as the immediate (Figure 5B) and late response to CDCA (Figure 5C). The initial increase in [Ca^2+^]_i_ was coupled with either no (Figure 5B, inset ^*^) or minimal (Figure 5B, inset ^*†*^) spatial movement. In contrast, later during the calcium response, some cells failed to display the spatial displacement (Figure 5C, inset ^*^), whereas others profoundly changed their location (Figure 5C, inset ^*†*^). The facts that (i) the [Ca^2+^]_i_ increase preceded the spatial displacement, and that (ii) [Ca^2+^]_i_ transients, similar to the ones in PDECs that did not displace, were recorded from all PDECs that underwent displacement, strongly substantiate that the observed [Ca^2+^]_i_ signals are not a motion artifact.

## Discussion

We successfully applied the tissue slice preparation technique to study PDECs. The main hallmark of the tissue slice approach, especially in contrast to the majority of studies that were done by isolating ducts using enzymes, is that the homotypic as well as heterotypic interactions are preserved. Histological and functional evaluations of the slices have shown that the slicing procedure did not damage the structure or the function of the tissue indicating that this technique represent an excellent *in situ* model in which the function and the cell-cell interactions of PDECs can be investigated.

For the study of pancreatic acini and islets, the thickness of the tissue slices is critical. The ideal thickness is 100–200 μm, depending on the use of the slice (Marciniak et al., 2014). For the functional cell imaging of PDECs, we found that a practically useful thickness of the slices is 140 μm, enabling the calcium dye to penetrate the cells and allowing for preservation of morphological tissue structure at the same time. Moreover, this thickness allowed for diffusion of gases and nutrients into the tissue. Tissue slices could be maintained for >8 h in HEPES-buffered solution at RT, however, for even longer studies optimization of the culturing media and use of culture incubators might be needed. One of the critical steps in the preparation of the slices was the injection of agarose. Since pancreas is a “soft” type tissue, injection of agarose serves as a scaffold that stabilized the tissue during cutting. The scaffold effect was achieved by injecting low-melting point agarose at 37°C into the common bile duct, filling the ductal tree retrogradely. Our results have shown that agarose reaches the head and the body of the pancreas and to a smaller extent the tail. We found that the injection procedure did not affect the function or the structure of the ductal cells as confirmed by the histological and functional investigations. Preservation of the intact epithelium has also been confirmed by the fact that the presence of the epithelial-specific ion channel, CFTR, could be detected on the apical membrane of the ductal cells.

Normal calcium signaling plays a central role in the physiological regulation of ${\text{HCO}}_{3}^{-}$ secretion by PDECs which is important for neutralization of protons secreted by acinar cells, as well as for keeping trypsinogen in an inactive form and washing it away. Pathologically changed calcium signals, through calcium overload of PEDCs, decreased ATP production due to mitochondrial damage, and impaired ${\text{HCO}}_{3}^{-}$ secretion seem to importantly contribute to pathogenesis of acute and chronic pancreatitis (Lee and Muallem, 2008). The toxic calcium signals may be an interesting therapeutic target and thus models that enable studies of PDECs function in normal and pathological conditions are of great practical relevance (Hegyi and Petersen, 2013; Maléth and Hegyi, 2014). To the best of our knowledge, there is only one previous study that analyzed calcium signals in response to bile acids in PDECs. In guinea pig intra-interlobular ducts, low concentration of CDCA, i.e., 0.1 mM, elicited regenerative calcium oscillations that lasted 2–5 min and at this concentration, ${\text{HCO}}_{3}^{-}$ secretion was significantly stimulated (Venglovecz et al., 2008). This concentration corresponds with concentrations of taurolithocholic acid sulfate (TLC-S) that elicited calcium responses in the majority of mouse acinar cells. Interestingly, the calcium response in acinar cells was qualitatively very similar to the one in guinea pig PDECs (Voronina et al., 2002). In contrast, 1 mM CDCA produced a transient increase in [Ca^2+^]_i_ lasting approximately 5 min, followed by a sustained plateau that returned to the baseline upon termination of stimulation. At this concentration, ${\text{HCO}}_{3}^{-}$ secretion was strongly inhibited (Venglovecz et al., 2008). High concentration of TLC-S (0.5 mM) caused a qualitatively very similar response in [Ca^2+^]_i_ in mouse acinar cells (Voronina et al., 2002). An important difference in the calcium response between the primary tissue and the slice preparation is that the transient was approximately an order of magnitude shorter (i.e., lasting about 30 s) in the case of slices. Also, in contrast to the behavior in acinar cells, the calcium signals did not seem to be synchronized between different PDECs (Petersen and Findlay, 1987). It needs to be pointed out however, that during supraphysiologically high [Ca^2+^]_i_ also acinar cells may be uncoupled (Hegyi and Petersen, 2013). In future studies, the dose dependence of calcium responses in PDECs needs to be studied into more detail and the slice preparation offers the possibility to simultaneously study the responses of acinar cells. In addition, the specific composition of mouse bile should be taken into account and different bile acids tested for their potential to produce regenerative or sustained calcium responses (Sayin et al., 2013). This shall enable us to assess whether the observed quantitative differences are due to different methodological approaches or due to inter-species differences in responses to bile acids, depending on the relevance of a given bile acid in a given species. Most importantly, a more detailed description of normal and pathological calcium signals in mouse PDECs can help us better understand the etiopathogenesis of pancreatitis and find new therapeutic targets.

An especially interesting observation in this study was that following the CDCA stimulation, we recorded movement of PDECs, a property of PDECs not shown before (Figure 5). This active PDEC movement was not uniformly detected in all the cells, moreover it was preceded by the [Ca^2+^]_i_ increase, confirming that the recorded [Ca^2+^]_i_ signal was not an artifact of this movement. It is not clear what is the mechanism causing the movement of PDECs following CDCA stimulation. A change in osmolality of the local milieu due to the stimulated ${\text{HCO}}_{3}^{-}$ secretion may result in an osmotically driven movement. However, we believe this not to be the case since (i) 1 mM CDCA more likely inhibits than stimulates ${\text{HCO}}_{3}^{-}$ secretion (Venglovecz et al., 2008), and (ii) the shape of the PDECs as well as of the surrounding acinar cells remained unaffected. More likely, myoepithelial cells in the ducts could provide a mechanistic substrate for active contraction (Puchtler et al., 1975). Therefore, further experiments will be needed to resolve this issue.

In conclusion, we have successfully applied the tissue slice preparation in which the structure and function of PDECs are preserved. This model represents an *in situ* microenvironment that enables studying PDECs under both physiological and pathophysiological conditions and their interaction with the acinar or endocrine cells. This model also opens up the possibilities to investigate human pancreatic function in an intact, *in vivo*-like environment.

## Data Availability

All datasets generated for this study are included in the manuscript and/or the Supplementary Files.

## Ethics Statement

Animal experiments were conducted in compliance with the Guide for the Care and Use of Laboratory Animals (United States, Department of Health and Human Services, NIH publication No 85-23, revised 1985) and the experimental protocol was approved by the local Ethical Board of the University of Szeged, the National Scientific Ethical Committee on Animal Experimentation (Budapest, Hungary), and the Veterinary administration of the Republic of Slovenia (permit number: U34401-12/2015/3).

## Author Contributions

EG was involved in all of the experiments and performed the Giemsa and CFTR staining. JD, AS, and VP performed the calcium imaging, analyzed and interpreted the data, and drafted and edited the manuscript. AÉ was involved in the Giemsa and CFTR staining. VV supervised the project and drafted the manuscript. All authors approved the final version of the manuscript.

### Conflict of Interest Statement

The authors declare that the research was conducted in the absence of any commercial or financial relationships that could be construed as a potential conflict of interest.

## Supplementary Material

Supplemental Video 1 XYZ-stack of OGB-1 loaded acute tissue slice. Morphology of the intralobular duct can be followed, and surrounding acinar and islet cells can be observed. Please note that the structure of acinar cells, as well as an islet of Langerhans, can also be observed in the XYZ-stack.

Supplemental Video 2 Video demonstrates construct of confocal time series images (see Results section for detailed description). CDCA stimulation from 3.07 s onwards. Background displays cellular morphology in gray, and an overlay of calcium response is depicted in green. See Results section for interpretation.

Supplemental Figure 1 OGB-1 loaded acute tissue slice demonstrating that different cells types can be visualized simultaneously. The yellow stars depict exocrine acinar cells, the yellow dashed line depicts the border of a pancreatic duct, and the yellow dashed-dotted line depicts the border of an islet of Langerhans.

Supplemental Figure 2 **(A)** OGB-1 loaded tissue slice. **(B)** On the same slice, PDECs responding to 1 mM CDCA (red) and spontaneously active acinar cells (blue) are depicted. Numbers correspond with calcium traces in **(C)**. **(C)** Calcium activity of PDECs during stimulation with 1 mM CDCA (red) and spontaneous activity of acinar cells within the slice shown in **(B)**. Numbers correspond with the labels in **(B)**.

## Abbreviations

CDCA: chenodeoxycholic acid
CFTR: cystic fibrosis transmembrane conductance regulator
ECS: extracellular solution
OGB-1: Oregon Green 488 BAPTA-1
PDECs: pancreatic ductal epithelial cells.
