# Supplementary figures and images for: A Novel in situ Approach to Studying Pancreatic Ducts in Mice
Source: Front Physiol. 2019 Jul 24;10:938. doi: 10.3389/fphys.2019.00938 (PMC6668154; doi:10.3389/fphys.2019.00938)

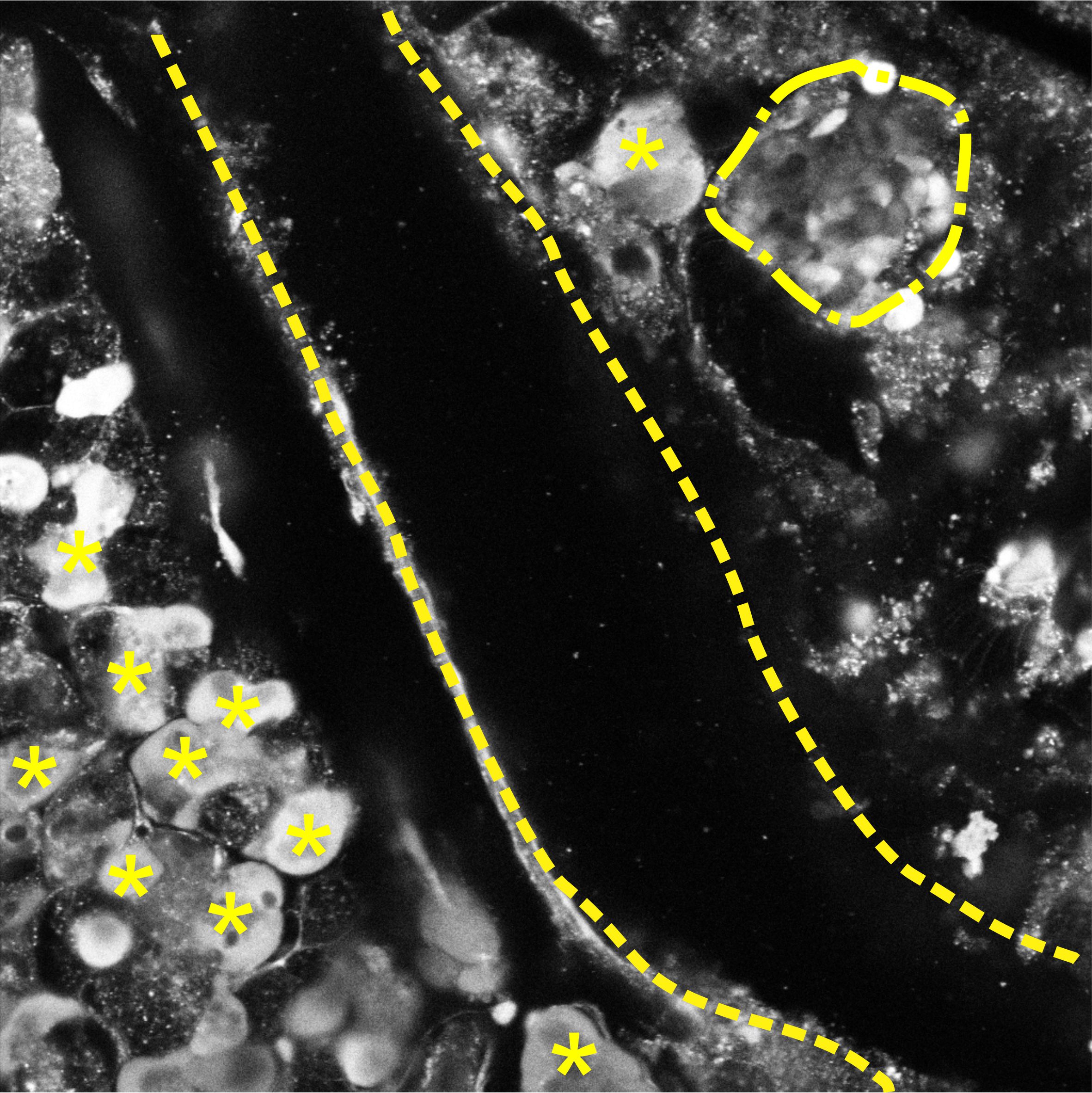

Supplement: Supplemental Figure 1 — OGB-1 loaded acute tissue slice demonstrating that different cells types can be visualized simultaneously. The yellow stars depict exocrine acinar cells, the yellow dashed line depicts the border of a pancreatic duct, and the yellow dashed-dotted line depicts the border of an islet of Langerhans. [file Image_1.JPEG]

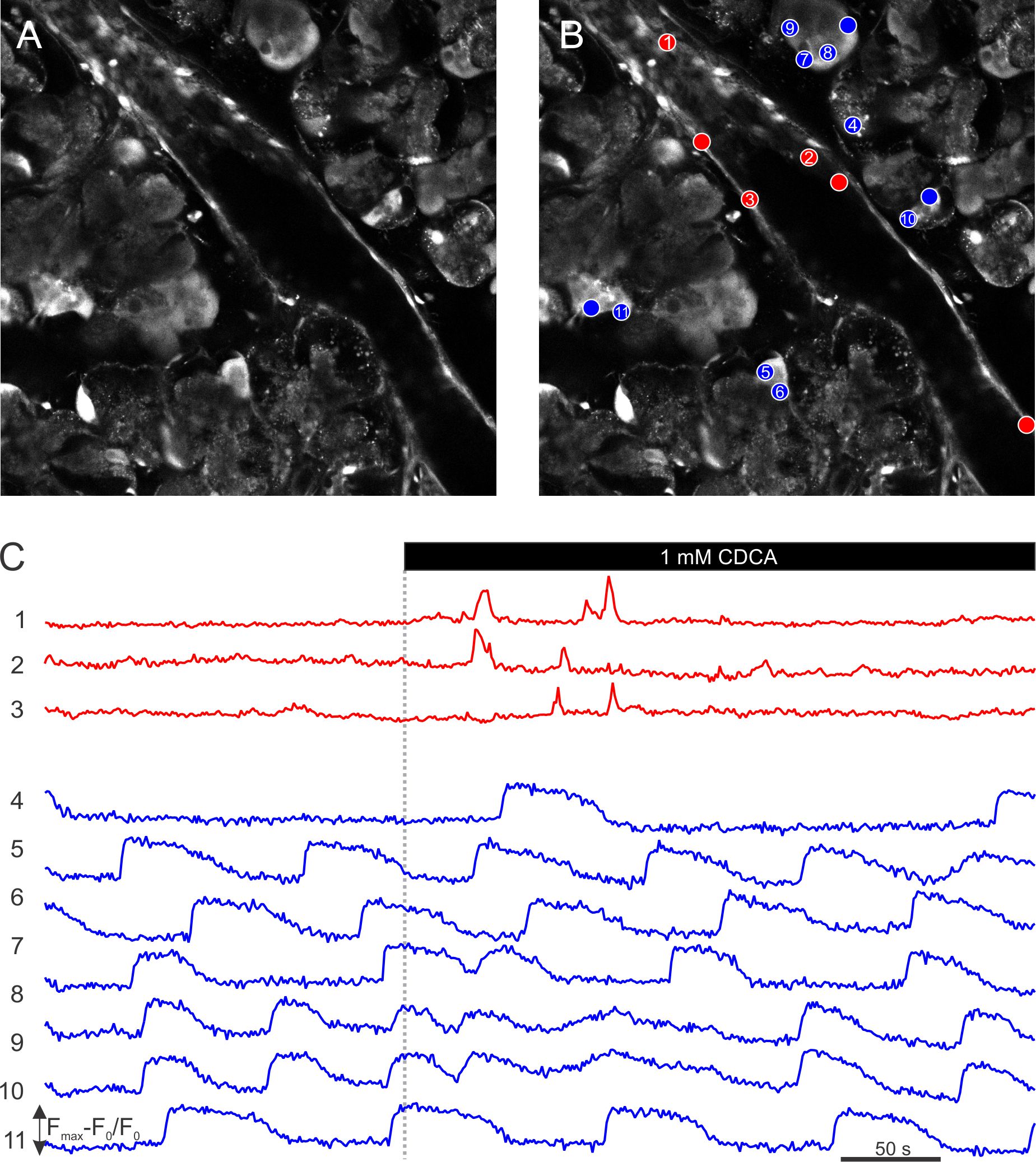

Supplement: Supplemental Figure 2 — (A) OGB-1 loaded tissue slice. (B) On the same slice, PDECs responding to 1 mM CDCA (red) and spontaneously active acinar cells (blue) are depicted. Numbers correspond with calcium traces in (C). (C) Calcium activity of PDECs during stimulation with 1 mM CDCA (red) and spontaneous activity of acinar cells within the slice shown in (B). Numbers correspond with the labels in (B). [file Image_2.JPEG]
